# Characterization of High-k Nanolayers by Grazing Incidence X-ray Spectrometry

**DOI:** 10.3390/ma7043147

**Published:** 2014-04-17

**Authors:** Matthias Müller, Philipp Hönicke, Blanka Detlefs, Claudia Fleischmann

**Affiliations:** 1 Physikalisch-Technische Bundesanstalt (PTB), Abbestr. 2-12, 10587 Berlin, Germany; E-Mail: philipp.hoenicke@ptb.de; 2 CEA Laboratory of Electronics and Information Technologies (LETI), Minatec Campus, 17 rue des Martyrs, 38054 Grenoble, France; E-Mail: blanka.detlefs@cea.fr; 3 Imec, Kapeldreef 75, BE-3001 Leuven, Belgium; E-Mail: fleischm@imec.be

**Keywords:** GIXRF, layer thickness, gate stack, reference-free analysis, ALD

## Abstract

The accurate characterization of nanolayered systems is an essential topic for today’s developments in many fields of material research. Thin high-k layers and gate stacks are technologically required for the design of current and future electronic devices and can be deposited, e.g., by Atomic Layer Deposition (ALD). However, the metrological challenges to characterize such systems demand further development of analytical techniques. Reference-free Grazing Incidence X-ray Fluorescence (GIXRF) based on synchrotron radiation can significantly contribute to the characterization of such nanolayered systems. GIXRF takes advantage of the incident angle dependence of XRF, in particular below the substrate’s critical angle where changes in the X-ray Standing Wave field (XSW) intensity influence the angular intensity profile. The reliable modeling of the XSW in conjunction with the radiometrically calibrated instrumentation at the PTB allows for reference-free, fundamental parameter-based quantitative analysis. This approach is very well suited for the characterization of nanoscaled materials, especially when no reference samples with sufficient quality are available. The capabilities of this method are demonstrated by means of two systems for transistor gate stacks, *i.e*., Al_2_O_3_ high-k layers grown on Si or Si/SiO_2_ and Sc_2_O_3_ layers on InGaAs/InP substrates.

## 1. Introduction

The development and fabrication of nanolayered gate stacks for nanoelectronic devices requires well-controlled deposition methods for high-k materials on various substrate materials and/or on substrates with different chemical character. Atomic Layer Deposition (ALD) allows for controlling the deposition at the atomic level, making use of the self-limiting chemisorption reactions. The layer thickness of the high-k material can be controlled by the number of ALD cycles applied. In the case of thick layer or bulk depositions the amount of deposited material per ALD cycle is constant because of the constant surface conditions for the next ALD cycle. However, during the early stages of the deposition on the initial substrate the growth rate can be lower than the steady-state growth rate of the bulk deposition [1,2]. One reason for a reduced initial growth rate could be that the starting surface is not fully reactive with the ALD precursors. In addition, island growth can occur due to nucleation of the ALD film at particular defect sites on the initial substrate [3,4].

For a well-controlled ALD of thin nanolayers we need a detailed understanding of the reaction mechanisms and chemistry. To achieve this, it is particularly important to reliably characterize the amount of deposited material during the initial stages of layer depositions, *i.e*., the first ALD cycles. The applicable analytical methods are, however, limited due to the low thickness of the high-k layer after a few cycles, which is typically below 1 nm. X-Ray Fluorescence analysis (XRF) is a highly sensitive technique to obtain elemental composition and mass deposition. In particular, XRF in grazing incidence geometry [5–9] allows for very low limits of detection.

In Grazing Incidence XRF (GIXRF), the incident angle between the X-ray beam and sample surface is varied around the critical angle for total external reflection. On flat samples, the interference between the incoming and the reflected beam results in an X-ray Standing Wave (XSW) field. The intensity distribution inside the XSW field strongly depends on the incident angle and can significantly enhance the emitted fluorescence intensity of an atom inside the XSW, while at the same time it reduces the substrate contribution. In contrast to conventional Total Reflection XRF (TXRF) at an angle of incidence fixed typically at 70% of the critical angle, the GIXRF technique can take advantage of the angular dependent XSW effect. Performing angular scans around the critical angle provide additional information about the depth distribution of the mass deposited on the substrate. The fluorescence signal of nanoparticles, thin layers, and implantation profiles show different angular dependencies [6] due to the varying XSW intensities, which enables elemental depth profiling by GIXRF [10].

The quantification methods of XRF rely on reference materials or calibration standards which are rarely available for a high variety of semiconductor materials. To overcome this lack of appropriate nanoscale reference materials [11,12] one can use reference-free (GI)XRF [13]. In this method, radiometrically calibrated instrumentation is used and no pre-calibration by calibration standards is required to obtain quantitative information. This enables a high flexibility of the technique in view of accessible materials, compositions and layer structures. Furthermore, the reference-free GIXRF can be used to qualify [14] calibration standards for, e.g., laboratory TXRF machines used in device processing for inline control.

In order to demonstrate the capability of reference-free GIXRF a set of Al_2_O_3_ high-k layers grown on Si or Si/SiO_2_ substrates was studied. These systems were chosen for their known differences in growth rates in the first ALD cycles, which are caused by the different reactivity of the H- and OH-terminated surface towards the precursor. Furthermore, we have investigated a rather unconventional material system, *i.e*., a Sc_2_O_3_ layer grown on InGaAs/InP substrates, to exemplify the high suitability of reference-free GIXRF for various material combinations.

## 2. Experiment and Samples

**Figure 1.**
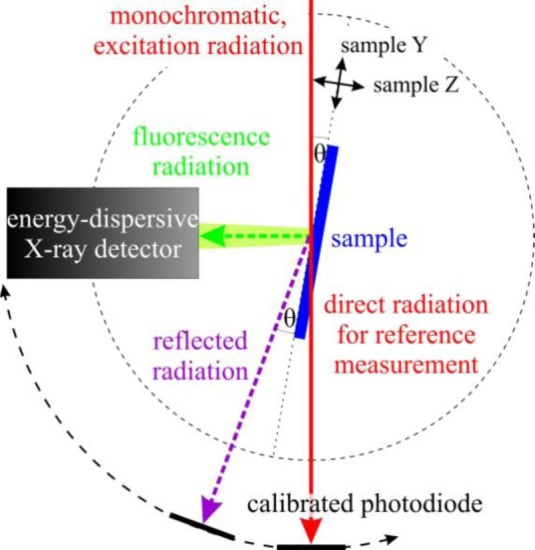
Sketch of the experimental GIXRF set-up in the UHV chamber.

The measurements were conducted employing the radiometrically calibrated instrumentation of the Physikalisch-Technische Bundesanstalt (PTB) and a fundamental parameter-based reference-free quantification approach [13,15]. An ultrahigh-vacuum (UHV) chamber [16] equipped with a 9-axis manipulator was used for the measurements allowing for very precise sample alignments in all relevant degrees of freedom. The incident angle θ between the X-ray beam and the sample surface can be varied between −5° and 110° with a resolution of 0.0005°, which is sufficient for the GIXRF experiments. Additional photodiodes on a 2θ axis allow for X-ray reflectometry measurements simultaneously to the GIXRF measurements. For the detection of the emitted fluorescence radiation, a silicon drift detector (SDD) mounted at 90° with respect to the incident beam and calibrated with respect to its detector response functions and detection efficiency [17] was used. The incident photon flux was monitored by calibrated photodiodes. A sketch of the set-up is shown in Figure 1.

The experiments were carried out at two different beamlines of PTB at the synchrotron radiation facility BESSY II (Helmholtz-Zentrum, Berlin, Germany). They cover an incident photon energy range between 78 and 10.5 keV, which is required to quantify the elements of interest in this study. In detail, the plane grating monochromator (PGM) beamline [18] for undulator radiation provides soft X-ray radiation of high spectral purity in the photon energy range of 78 eV to 1860 eV, which allows for the excitation of fluorescence emission from the Al-K shell (1559 eV). To excite fluorescence emission from the Sc-K shell (4492 eV) hard X-ray radiation between 1.75 and 10.5 keV is available at the four-crystal monochromator (FCM) beamline [19] using bending magnet radiation.

The sample sets investigated in this work are listed in Table 1 together with the respective cycle numbers used for the ALD deposition. Set 1 comprises Al_2_O_3_ layers grown on Si substrates that are cleaned in HF solution (ID1–ID3) or accompany a chemically grown SiO_2_ layer (ID4–ID7). In the second set, InP substrates with a 300 nm InGaAs layer are used, onto which Sc_2_O_3_ and Al_2_O_3_ layers have been deposited (ID8–ID11).

Each sample was aligned with respect to the incoming X-ray beam in order to ensure a well defined geometry. This is crucial for the incident angle between the X-ray beam and the sample surface. This angle was then varied in small steps from 0° to angles beyond the critical angle for total external reflection, recording the emitted fluorescence radiation with a typical integration time between 30 s and 90 s.

## 3. Reference-Free Quantification

The knowledge of all relevant instrumental and fundamental parameters [20,21] allows for calculating [15,22] the mass deposition *m_i_*/*F*_1_ of the element *i* with unit area *F*_1_:

$$\begin{array}{l} \frac{m_{i}}{F_{1}}=-\frac{1}{\mu_{\text{tot,}i}}\ln\left\{1-\frac{P_{i,j}}{P_{\text{in}}I_{\text{XSW}}(E_{\text{in}},\theta )\frac{\Omega_{\text{det }1Q_{i}(E_{\text{in}})}}{4{\pi \;\sin\theta \;\mu }_{\text{tot},i}}}\right\},\text{with}\\ \mu_{\text{tot},i}=\frac{\mu_{i}(E_{\text{in}})}{\sin\theta }+\frac{\mu_{i}(E_{i,j})}{\sin(90{}^{\circ}-\theta )}\text{and}\;Q_{i}(E_{\text{in}})=\tau_{Xi}(E_{\text{in}})\omega_{Xi}g_{j,Xi} \end{array}$$ (1)

Here θ is the glancing angle, *E*_in_ is the photon energy of the incident (excitation) radiation, *P*_in_ is the photon flux (photons per second) of the incident radiation, *I*_XSW_ is the relative intensity of the exciting radiation within the layer modulated by the XSW, *E_i,j_* is the photon energy of the emission line *j* of the element *i*, τ*_Xi_*(*E*_in_) is the photoelectric cross section of the element *i* at the photon energy *E*_in_, μ*_i_*(*E_i,j_*) is the absorption cross section of the element *i* at the photon energy *E_i,j_*, Ω_det_ is the effective solid angle of detection [22], ω*_Xi_* is the fluorescence yield of the absorption edge *Xi* (of the element *i*), and *g_j,Xi_* is the transition probability of the emission line *j*.

**Figure 2.**
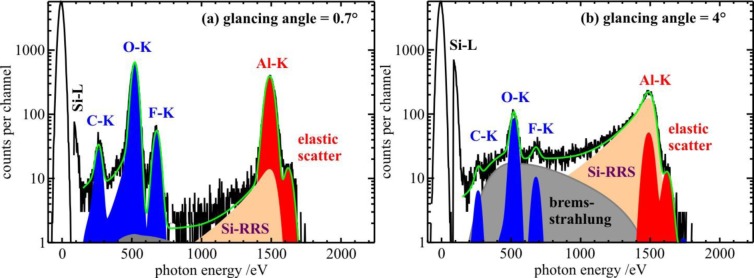
Spectra of a thin Al_2_O_3_ layer on a Si substrate (sample ID1) excited by a 1622 eV X-ray beam under a glancing angle of 0.7° (**a**) and 4.0°; (**b**) at the PGM beamline. The deconvolution of the spectra was performed by fitting a model spectrum (green line) employing the detector response functions. The fluorescence lines as well as the background contributions of Bremstrahlung and Resonant Raman Scattering (RRS) are marked in different colors.

**Figure 3.**
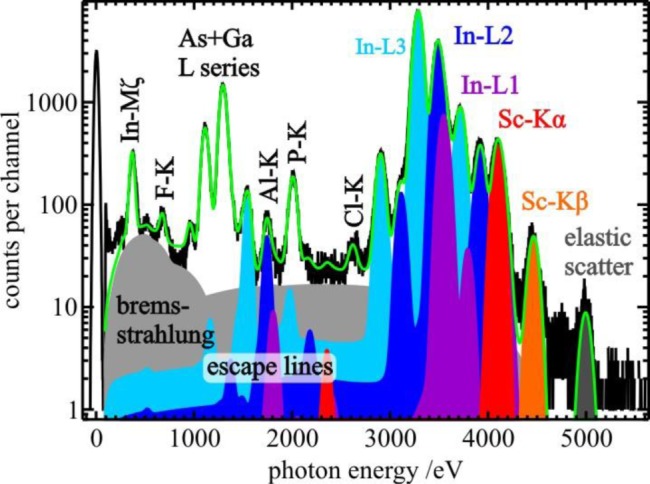
Fluorescence spectrum (black line) of the sample ID8 with one ALD cycle for Sc_2_O_3_ excited by a 5 keV X-ray beam under a glancing angle of 0.5° at the FCM beamline. The deconvolution of the spectrum was performed by fitting a model spectrum (green line) employing the detector response functions. The indium fluorescence line series were fitted using constant line sets [23] for each sub-shell (light blue, blue and purple).

The fluorescence intensity *P_i,j_* is the count rate of emission line *j* divided by the detection efficiency of the SDD detector for the photon energy *E_i,j_* of the respective emission line and represents the photon flux of the emitted fluorescence radiation on the active area of the detector (photons per second). A spectral deconvolution procedure was used to obtain the count rates of each line, as is exemplified in Figures 2 and 3, for sample ID1 and ID8, respectively. This procedure fits a model spectrum to the measured spectrum. The model spectrum consists of single fluorescence lines, constant fluorescence line sets [23] for the three In-L sub shells (high-energy spectra only), and background contributions like Bremsstrahlung produced by photo-electrons, as well as resonant Raman radiation [24,25]. In addition, the model spectrum was convoluted by the detector response functions in order to take into account instrumental line broadening and artifacts in the spectrum like escape lines, shelf, and tailing [17]. The life time of the spectra was derived from the zero-energy peak, which was generated by a 1 kHz electronic pulser.

Figure 2 shows two spectra and the respective model spectra of sample ID1 (3 Al_2_O_3_-ALD cycles on Si substrate) excited well above the Al-K shell binding energy. The photon energy of the incident X-ray beam was tuned to 1622 eV and a glancing angle of (a) 0.7° and (b) 4.0° was used. The critical angle of total reflection for X-rays of 1622 eV on Si and SiO_2_ is about 0.9°. Spectrum (a), which is recorded below the critical angle, shows a drastically higher signal-to-background ratio of Al compared to the spectrum (b) of the same sample taken well-above the critical angle, illustrating the advantage of TXRF geometry for trace element analysis [6,22]. Figure 3 shows the spectrum and the related fitting results of sample ID8 (1 Sc_2_O_3_− and 1 Al_2_O_3_-ALD cycle on InGaAs). Here, the glancing angle was tuned to 0.5° which is the critical angle of total reflection for a 5 keV X-ray beam on the InGaAs/InP substrate.

It is clear that at low glancing angles the sensitivity for the deposited material is higher due to the lower spectral background generated by the substrate, independent of the material system. In order to evaluate the reliability of the quantification method at these low glancing angles we have modeled, using the IMD software package of Windt [26] (New York, NY, USA), the enhancement of the fluorescence intensity by the XSW inside the layer to be quantified. With this, we calculated the relative intensity *I*_XSW_ as a function of the glancing angle and depth with respect to the surface. The thickness of the Al_2_O_3_ and Sc_2_O_3_ layers used for the XSW calculation was derived from spectra measured at high glancing angles (above 1.5°). Here, the XSW effect is negligible because of the low intensity of the reflected beam. For better clarity the quantified mass deposition was converted into a layer thickness using the tabulated bulk density [27] of Sc_2_O_3_ (3.86 g/cm^3^) and Al_2_O_3_ (4.00 g/cm^3^). Due to the low thickness and the deposition process of the layers their actual density may differ from the bulk density [28], which would affect the calculated layer thickness. The impact of the layer thickness on the XSW calculation for glancing angles is discussed in section 4.2. In the case of incident angles above 1° the uncertainty resulting from differences (up to 25%) in the layer density (thickness) is less than 0.5%. Using the calculated XSW intensities, the quantification of the Sc_2_O_3_ mass deposition was repeated for all glancing angles to validate the accuracy of the XSW calculation.

For the quantification of the Al_2_O_3_ high-k layers of both sample sets, *i.e*., Si and InGaAs, the measurements at a glancing angle of 4° were used for which the effect of the XSW in negligible. This approach is only applicable if the mass deposition of interest is well above the lower limit of detection at incidence angles well above the critical angle of total reflection.

The relative uncertainties of the determined mass depositions are mainly given by the estimated uncertainties of the fundamental parameter data [13,29] used for the calculation of the fluorescence intensity. The fundamental parameters used in this work result in a relative uncertainty of 11% for the Al-Kα and 7% for Sc-Kα radiation. The calculation of the XSW contributes only with a relative uncertainty of 0.6% to the Sc_2_O_3_ quantification due to the low enhancement (<12%) above 1° incident angle. The third source of uncertainty is the instrumentation. The most relevant instrumental parameter for the uncertainty budget of the reference-free quantification is the effective solid angle of detection, which is calculated from the active detector area, the distance of the detector to the sample, the width of the incident beam and the angle of incidence. Summing up all uncertainty contributions the relative uncertainty of the effective solid angle of detection is 4.0%. The total relative uncertainty of all other instrumental parameters is 2.0%. The determination of the count rates by the spectral deconvolution contributes with a relative uncertainty of 5.0% and the counting statistic with up to 1.8% (Sc) and 2.4% (Al) to the quantification.

All uncertainty contributions sum up to a total relative uncertainty of the reference-free quantification of 13% for Al and 10% for Sc. There is no significant difference in the relative uncertainties of the Al quantification for the different sample sets.

## 4. Results and Discussion

### 4.1. Sample Set 1: Al_2_O_3_ High-k Nanolayer on Si and SiO_2_

The mass depositions of the Al_2_O_3_ layers resulting from the reference-free quantification are listed in Table 2. A significant difference in the deposited mass on the Si and SiO_2_/Si substrates was observed during the first cycles. These results can be used to derive growth rates and investigate differences in chemical processes governing the layer deposition. The general trend in the data observed between the HF treated Si surface (H-terminated) and the Si substrate with a chemically grown oxide (OH-terminated) is in agreement with previous reports [3,30], and reflects the difference in their chemical reactivity towards the ALD precursor. It was shown previously that the lower reactivity of the H-terminated surface towards the precursor compared to the oxidized Si surface may lead to growth inhibition, which has been assigned to island growth behavior. In general, growth inhibition may persist up to 20 cycles [31] or more.

**Figure 4.**
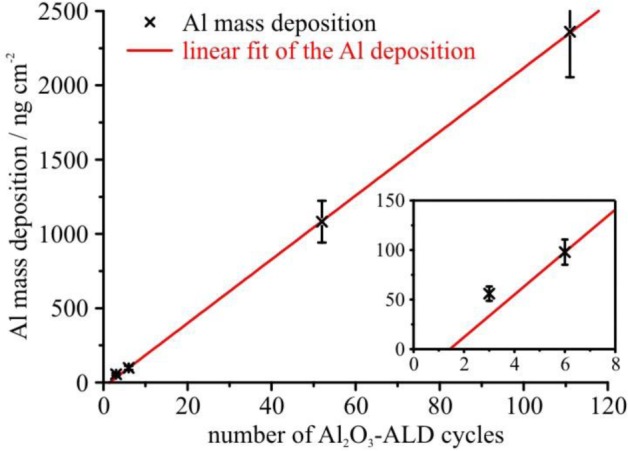
Al mass deposition on SiO_2_ of the samples ID4–ID7 (sample set 1) obtained by reference-free GIXRF at a glancing angle of 4°.

The data obtained on the SiO_2_ samples (ID4-ID7) show a linear increase in the mass deposition with the number of ALD cycles after the 6th cycle. With a linear fit to these data (see Figure 4) the growth rate can be determined, which amounts to 21.5 ng/cm^2^ per ALD cycle. For the initial cycles a slight deviation from this growth behavior can be observed, as is visible in the inset to Figure 4. A linear fit to these initial data points yields a growth rate of 16.3 ng/cm^2^ per cycle, which is significantly lower. This result indicates some initial growth inhibition on the oxidized Si surface. Whether an island growth mechanism is the origin of this growth behavior, similar to what was reported for the HF last surface, cannot be verified with the study presented here due to a lack of data points and complementary analysis. However, this observation clearly demonstrates the high sensitivity of the reference-free GIXRF method. Relying on a well-known reference system (Al_2_O_3_ on Si), these findings proof and validate the capabilities of reference-free GIXRF. At the same time, they highlight the importance of highly sensitive and reliable characterization methods for the study of growth mechanisms in these systems. An in-depth analysis and discussion of the ALD deposition process and gas-solid reactions on Si substrates can be found in [3].

### 4.2. Sample Set 2: Sc_2_O_3_ High-k Nanolayer on InGaAs

**Figure 5.**
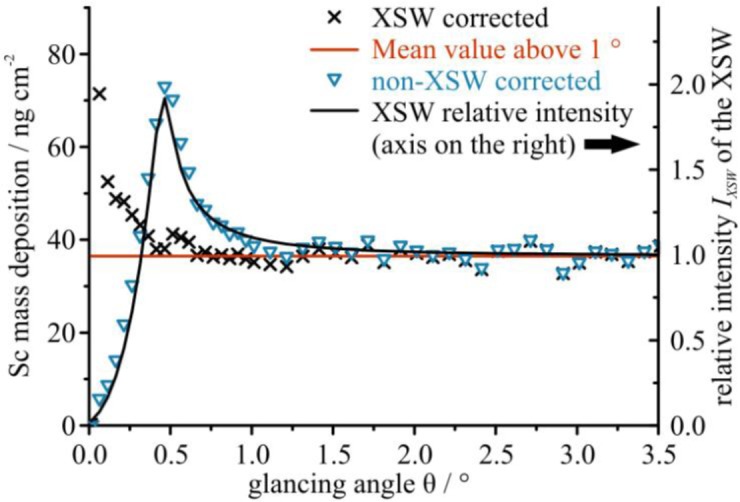
Plot of the Sc mass deposition (sample ID8) quantified for each glancing angle with (×) and without (∇) taking into account the XSW enhancement calculated in a non-iterative way as described in Section 3. In addition, the relative intensity of the XSW is plotted on the right axis (black line).

For the Sc_2_O_3_ and Al_2_O_3_ layers on InGaAs the quantification was performed for each glancing angle of the GIXRF scan ranging from 0° up to 3.5°. The critical angle for 5 keV X-rays reflected on an InGaAs substrate is approximately 0.5°. In Figure 5 the determined mass deposition of Sc is plotted over the glancing angle. The quantification was performed with and without taking the XSW into account to highlight the impact of the XSW enhancement.

The results without consideration of the XSW (Figure 5, open triangles) show a mass deposition which varies with the glancing angle, following the slope of the relative intensity of the XSW within the Sc_2_O_3_ layer (Figure 5, solid line). Taking into account the XSW enhancement (Figure 5, crosses) the mass deposition is constant and remains independent of the incident angle (for angles above 0.3°). The increase in mass deposition observed at very low glancing angles can be caused by the layer model used for the XSW calculations. This model was derived from the initial quantification at high glancing angles with the assumption that the Al_2_O_3_ and the Sc_2_O_3_ layers are well separated, continuous layers with no intermixing or alloy formation and nominal bulk densities. Essentially, the employed Al_2_O_3_ densities, which determine the thickness when the mass deposition of a layer is given, have a strong impact on the XSW calculation for low glancing angles (<1.0°). In the present case we found deviations of up to 13% for the XSW intensities within the Sc_2_O_3_ layer for angles below 0.5° and a 20% reduction of the Al_2_O_3_ density. The impact on the XSW intensity within the Al_2_O_3_ layer is much lower, e.g., in the example above we found a change of less than 5%. For an improved quantification, an iterative modeling that optimizes the layer structure including the densities together with the quantification is required. Such quantification methods already exist for depth profiling of ion implantations in silicon [32] and are currently under development for the characterization of thin layered samples. Besides an incorrect assumption in the layer model, the uncertainty of the effective solid angle of detection, which is higher at lower glancing angles, can likewise have an impact on the results obtained for angles below 0.3 deg.

To quantify ultra-low mass depositions, measurements in TXRF geometry at 70% of the critical angle (here 0.35°) would be favorable to improve the signal-to-background ratio. In the case of the thinnest Sc_2_O_3_ layer (1 ALD cycle) the lower limit of detection for Sc on InGaAs is increasing with increasing glancing angles as follows: 3.2 pg/cm^2^ at 0.35°, 4.1 pg/cm^2^ at 0.5°, 15.2 pg/cm^2^ at 1.5° and 22.6 pg/cm^2^ at 3.5°.

**Figure 6.**
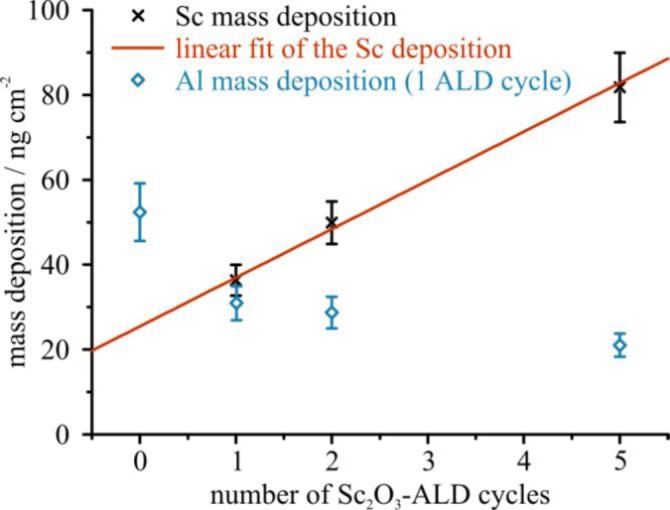
Quantified Sc and Al mass deposition plotted over the number of Sc-ALD cycles.

Table 3 provides the Sc mass depositions quantified by taking into account the XSW calculations, using the Al mass depositions obtained by soft X-ray GIXRF measurements at a 4° glancing angle (see discussion above). The listed results are the mean values of the Sc mass deposition derived for all glancing angles above 1.0°. Performing a linear fit of these results gives a growth rate of 11.4 ng/cm^2^ per Sc_2_O_3_-ALD cycle (see Figure 6).

The quantification of the Al_2_O_3_ top layer shows a slightly decreasing Al mass deposition with increasing Sc_2_O_3_ thickness in the layer below. This indicates a lower Al_2_O_3_ growth rate on the Sc_2_O_3_ surface than on the InGaAs surface. This is supported by the result obtained on sample ID11, which shows the highest mass deposition for one Al_2_O_3_-ALD cycle on a pure InGaAs surface. For this sample, the Al_2_O_3_ layer was deposited directly on the InGaAs surface while the Sc_2_O_3_ layer was deposited subsequently on top of the Al_2_O_3_ layer. Note, however, that this assumption needs to be verified by a more systematic study utilizing a larger number of samples. For more details about the growth mechanisms and layer properties we refer to [33]. Our results clearly demonstrate the flexibility of our method in view of material systems and compositions.

## 5. Conclusions

The results presented in this study demonstrate the capability of the reference-free GIXRF measurements to quantify low mass depositions of a high variety of elements on various substrate materials. This is the prerequisite for (fundamental) studies aiming at a detailed understanding of deposition processes, reaction mechanisms and origins of growth inhibition. In addition to its fundamental importance, the reference-free quantification method is also of high relevance in process technology and layer engineering, where quantification of various materials is performed on a routine basis relying on calibration standards. The reference-free GIXRF method allows for the qualification of calibration standards used for laboratory equipment or to perform a reliable quantification of rather unconventional materials. This quantification approach reduces the dependency on appropriate reference materials, which are rarely available for nanolayered systems and enables the qualification of calibration standards for inline TXRF analysis.

Relative uncertainties of the analytical results in the range of 10% to 13% were achieved and are mainly limited by the utilized fundamental parameters. If the effect of the XSW is low, all other uncertainty contributions are about 5%. It was demonstrated that the calculation of the XSW, using available software packages and a rather simple non-iterative modeling of the layer structure, can correct the quantification results for glancing angles below the critical angle, but significant discrepancies were observed below 70% of the critical angle. If necessary, the contribution of the XSW field calculation to the total experimental uncertainty could be further reduced by using an iterative modeling for the XSW calculation and using also the X-ray reflectivity signal of each sample.

## Figures and Tables

**Table 1.** Description of the characterized Atomic Layer Deposition (ALD) samples.

ID	ALD layers	Comment	Layer structure
**sample set 1 (Si substrate)**			
1	3 cycles Al_2_O_3_	HF last	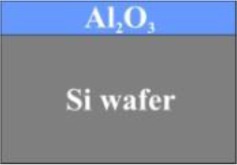
2	6 cycles Al_2_O_3_	HF last	
3	9 cycles Al_2_O_3_	HF last	
4	3 cycles Al_2_O_3_	chemical SiO_2_	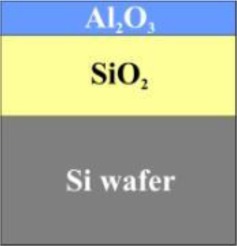
5	6 cycles Al_2_O_3_	chemical SiO_2_	
6	52 cycles Al_2_O_3_	chemical SiO_2_	
7	111 cycles Al_2_O_3_	chemical SiO_2_	
**sample set 2 (300 nm InGaAs on InP substrate)**			
8	1 cyc. Sc_2_O_3_ + 1 cyc. Al_2_O_3_	after HCl pre-clean	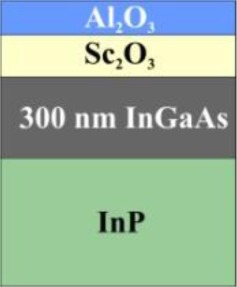
9	2 cyc. Sc_2_O_3_ + 1 cyc. Al_2_O_3_	after HCl pre-clean	
10	5 cyc. Sc_2_O_3_ + 1 cyc. Al_2_O_3_	after HCl pre-clean	
11	1 cyc. Al_2_O_3_ + 1 cyc. Sc_2_O_3_	Al_2_O_3_-ALD before Sc_2_O_3_	

**Table 2.** Mass depositions and high-k layer thicknesses of sample set 1 determined by Grazing Incidence X-ray Fluorescence (GIXRF) at a glancing angle of 4°. For better clarity, the respective mass deposition was transferred into a layer thickness using the bulk density and a 2 to 3 stoichiometry.

Sample ID	Number of Al_2_O_3_-ALD cycles	Comments	Al mass deposition (ng/cm^2^)	Al_2_O_3_ thickness (nm)
1	3	HF last (Si)	7.4 ± 1.0	0.036
2	6	HF last (Si)	16.7 ± 2.2	0.080
3	9	HF last (Si)	35.6 ± 4.7	0.17
4	3	chemical (SiO_2_)	56.0 ± 7.3	0.27
5	6	chemical (SiO_2_)	97.9 ± 12.8	0.47
6	52	chemical (SiO_2_)	1080 ± 140	5.2
7	111	chemical (SiO_2_)	2400 ± 310	11.4

**Table 3.** Mass deposition and high-k layer thickness of sample set 2 determined by GIXRF. For better clarity, the respective mass deposition was transferred into a layer thickness assuming the bulk density and a 2 to 3 stoichiometry.

Sample ID	Number of Sc_2_O_3_-ALD cycles	Sc mass deposition ^*^ (ng/cm^2^)	Sc_2_O_3_ thickness (nm)	Number of Al_2_O_3_-ALD cycles	Al mass deposition ^#^ (ng/cm^2^)	Al_2_O_3_ thickness (nm)
8	1	36 ± 4	0.14	1	31 ± 4	0.15
9	2	50 ± 5	0.20	1	29 ± 4	0.14
10	5	82 ± 8	0.32	1	21 ± 3	0.10
11	1 (on top of Al_2_O_3_)	29 ± 3	0.11	1 (deposited on InGaAs)	52 ± 7	0.25

* value of the mass deposition quantified at glancing angles ranging from 1.0° to 3.5° including the XSW correction;

# measured at a glancing angle of 4°.
